# Sensory symptoms associated with autistic traits and anxiety levels in children aged 6–11 years

**DOI:** 10.1186/s11689-024-09562-9

**Published:** 2024-08-12

**Authors:** Peter Bang, Danait Kidane Andemichael, Johan F Pieslinger, Kajsa Igelström

**Affiliations:** Division of Cell and Neurobiology, Department of Biomedical and Clinical Sciences, Linköping University, Linköping, 58185 Sweden

**Keywords:** Broad autistic phenotype, Central auditory processing disorder, Developmental Coordination Disorder Questionnaire, Dimensional measures, Glasgow sensory questionnaire, Hyperacusis, Research Domain Criteria

## Abstract

**Background:**

Autism spectrum conditions (ASC) and quantitative autistic traits (QATs) are associated with sensory symptoms, which may contribute to anxiety and adversely affect social and cognitive development. Although sensory symptoms can occur across all senses, the relative roles of specific sensory modalities as contributors to the autistic phenotype and to anxiety are not well understood. The objective of this study was to examine which sensory symptoms were most predictive of high anxiety.

**Methods:**

We recruited 257 female primary caregivers of children aged 6 to 11 years (49% girls) to a questionnaire study comprising parent-report measures for classical QATs (social, communicative, and rigid), autism-related sensorimotor symptoms (visual, auditory, tactile, olfactory, gustatory, vestibular, proprioceptive, and motor), and anxiety symptoms. First, Bayesian stochastic search variable selection (SSVS) was used to identify the most probable sensorimotor predictors of specific QATs as well as diagnosed ASC. Then, the selected predictors were used in another SSVS, using anxiety symptoms as a dependent variable, to identify which of the autism-relevant sensorimotor symptoms were most robustly predictive of anxiety. Finally, the effect sizes of anxiety-related sensory symptoms were estimated with linear regressions.

**Results:**

We found that auditory symptoms and motor difficulties were most predictive of ASC diagnosis. Developmental motor difficulties were also strongly related to all individual QATs, whereas auditory symptoms were more selectively predictive of rigid traits. Tactile symptoms robustly predicted social interaction QATs, and proprioceptive symptoms predicted communicative QATs. Anxiety outcomes were most strongly predicted by difficulties with auditory and olfactory processing.

**Conclusions:**

The results support the clinical importance of being alert to complaints about sounds and hearing in neurodevelopmental populations, and that auditory processing difficulties may be evaluated as an early marker of poor mental health in children with and without diagnosed autism. Olfactory processing differences appeared to be an anxiety marker less strongly associated with ASC or QATs, while motor difficulties were highly autism-relevant but not equally strongly associated with anxiety outcomes. We suggest that future studies may focus on the mechanisms and consequences of neurodevelopmental central auditory processing dysfunction and its potential relationship to anxiety disorders.

## Background

Autistic children are at high risk of anxiety, which begins early in life and increases with age [1, 2]. Anxiety can cause withdrawal, lead to suboptimal coping strategies such as self-harm, and significantly compromise quality of life [3, 4]. Children and adults with high, subclinical, quantitative autistic traits (QATs) also have an elevated risk of anxiety disorders [5, 6], and there is a dimensional relationship between anxiety levels and QATs at ages ranging from childhood to old age [7–10]. Anxiety in people with high QAT may in part be a consequence of social defeat, intolerance of uncertainty, or sensory unpredictability [9, 11], but trait anxiety may also play a role [12].

The neurobiological underpinnings of anxiety in autism are not understood [13], but one of the most consistent correlates is sensory over-responsivity [1]. Longitudinal research has suggested that sensory differences occur prior to anxiety in young children, potentially contributing to its development [14]. Sensory over-responsivity is also common and predictive of anxiety in non-autistic children [15]. Further, children’s scores on the parent-completed Glasgow Sensory Questionnaire (GSQ) were correlated with both anxiety scores and QATs [16]. Sensory sensitivity is starting to be regarded as a QAT [17], as it seems to share genetic underpinnings with classical QATs [17–19]. It appears likely that there are multiple complex bidirectional relationships between classical QATs, sensory differences, and anxiety.

Classical QATs comprise sociocommunicative and cognitive difficulties characteristic of autism. Typical social and communicative differences include low or atypical engagement with other people, and difficulties with using or understanding non-verbal and social pragmatic communication. In the non-social domain, differences include rigid or repetitive behaviors, special interests, and an intolerance to change or uncertainty. QATs within these three core domains can be measured with subscales of commonly used instruments, such as the Broad Autism Phenotype Questionnaire (BAPQ), Autism Spectrum Quotient (AQ), or Comprehensive Autistic Traits Inventory (CATI) [17, 20, 21]. The first classical QAT is related to social interaction and reflects decreased social motivation and difficulties obtaining or maintaining relationships. The BAPQ measures this construct in the subscale Aloofness, whereas the CATI and AQ do it with subscales for Social Interaction/Skills. The second QAT corresponds to the domain of non-verbal communication and pragmatic language skills, and is measured by the Pragmatic language subscale of the BAPQ or Communication subscales of the CATI/AQ. The social interaction and communication QATs also correspond to two of the components of the classical autism triad and are reflected in past and present diagnostic criteria. The third QAT is rigid personality, with resistance to change and desire for sameness. Rigid QATs are measured by the Rigid personality subscale of the BAPQ, the Cognitive Rigidity subscale of the BAPQ, or the Attention Switching subscale of the AQ. The modern CATI further measures Repetitive Movements and Sensory sensitivities as separate QAT factors under the domain of rigid/repetitive behaviors (RRB). The older AQ questionnaire contains subscales for Imagination and Attention to Detail, derived from original factor analyses, with less obvious parallels to the diagnostic domains. The social, communicative and rigid QATs have been suggested to be a “fractionable triad”, a constellation of traits with separate underpinning that vary independently from each other [22].

Whereas autistic populations have high scores across the triad, the independence of QATs is more obvious in high-risk individuals as well as in the general population [23]. Therefore, mechanisms associated with one QAT may be irrelevant to another, making it meaningful to study the QAT domains in isolation from each other. Similarly, many previous studies have conceptualized autistic sensory differences in terms of Dunn’s model of sensory processing, distinguishing between the axis of self-regulation (seeking/avoidance) and sensory thresholds (sensitivity/low registration), or as multimodal hyper- and hypo-responsiveness [e.g., 18, 19, 24, 25], with less emphasis on the potential differences between sensory modalities associated with segregated systems for early processing in the brain. In a recent study, we studied the relationships between sensory differences and QATs, fractionating not only the classical triad (using the BAPQ) but also sensory symptoms [26]. In contrast to most previous work, we used subscale scores for seven sensory modalities (Glasgow Sensory Questionnaire, GSQ; 24) instead of total sensory symptoms, and used Bayesian methods across two large adult samples to identify the most robust phenotypic relationships. We found that (1) auditory symptoms strongly predicted all three classical QATs, (2) proprioceptive symptoms predicted communicative QATs, and (3) tactile symptoms predicted social QATs.

This work raised several new questions, which the current study aimed to answer. The first question was whether modality-specific relationships would replicate in children. If they did, it would strengthen the case for their relevance to neurodevelopmental disorders and possible genetic underpinnings. Secondly, the relationship between proprioception and communicative QATs was difficult to interpret because it might have been confounded by the inclusion of items probing motor function in the proprioception subscale on the GSQ. Therefore, it is important to include measures of developmental coordination difficulties to control for that. Third, the potential relevance of the relationships to mental health was not tested. Our aim was to answer these questions by replicating and extending the analysis in a pediatric sample, using parent report of sensory and motor functions, QATs, and anxiety levels.

## Methods

### Participants and recruitment

The participants were primary caregivers of children aged 6 to 11 years. We chose to avoid confounding effects of informant sex by recruiting only female caregivers. Participants were recruited from Prolific.co, using the following preselection filters: female with one or more children, fluent English, having completed at least secondary education (GED/GCSE), and a good Prolific record (minimum 5 previous submission, minimum 99% approval rate). More detailed data on socioeconomic status or education were not recorded. The child had to have normal or corrected-to-normal vision, not have a known hearing impairment, and not have a serious or long-term physical illness. The study received 277 responses for children within the eligible age range (6–11 years). Two children were excluded due to cerebral palsy, one due to Down’s syndrome, one due to deafness, and one due to the parent failing an attention check. After removing participants with missing responses, 257 participants remained. As a way of enhancing representation of higher QATs in the sample, we recruited the final 20% of the sample using an additional filter, including parents who had a diagnosis of autism, were waiting to be evaluated for autism, or who self-identified as autistic. The two recruitment paths led to the same questionnaire and participants were immediately pooled for all analyses. The final sample included 10 autistic mothers and 31 mothers who reported suspected or self-identified autism, and 10 of these participants reported that their child had an autism diagnosis.

### Measures

#### Demographic questions

We asked about the relationship of the informant to the child (biological mother, adoptive mother, or other), age of the child, gender of the child, country of residence, neurodevelopmental disorders, primary sensory deficits, and other serious/long-term disorders. We also asked about diagnosed autism spectrum conditions (ASC) in the informant (if biological parent) or other first-degree relative, as a measure of genetic load.

#### Autism quotient – child version

The Autism Spectrum Quotient—Children’s Version (AQ-Child) is a parent-report measure designed to measure QATs in children aged 4 to 11 years [27]. The scale is similar to the adult AQ except three items have been excluded (items 29, 30, 49). The 47 items are rated on a 4-point Likert scale (0 = Definitely Agree, 1 = Slightly Agree, 2 = Slightly Disagree, 3 = Definitely Disagree, or the opposite for reverse-scored items). We used the original social, communicative, and rigid subscales (Social Skills, Communication, and Attention Switching), rather than subscales from the factor analysis performed by Auyeung et al. [27], as they more closely resembled the constructs used in our previous study (BAPQ’s Aloof personality, Pragmatic language deficits, and Rigid personality). The internal consistencies were high (Table 1). We used the original Communication subscale instead of the proposed mind reading subscale [27], because the latter included several rigid/repetitive items. These choices ensured that results would be comparable with the previous adult study. Table 1. Internal reliabilities of scales and subscales.

**Table 1:** Internal reliabilities of scales and subscales

	Cronbach’s α (95% CI)
Autism Spectrum Quotient	
Total	0.949 (0.940–0.957)
Social skills (Social QAT)	0.882 (0.860–0.902)
Communication (Communicative QAT)	0.859 (0.831–0.883)
Attention switching (Rigid QAT)	0.837 (0.805–0.865)
Glasgow Sensory Questionnaire	
Total	0.959 (0.952–0.966)
Visual	0.820 (0.784–0.850)
Auditory^a^	0.826 (0.790–0.857)
Tactile	0.740 (0.688–0.785)
Olfactory	0.730 (0.676–0.778)
Gustatory^a^	0.748 (0.704–0.787)
Proprioceptive	0.842 (0.811–0.869)
Vestibular	0.772 (0.725–0.812)
Developmental Coordination Disorder Questionnaire	0.932 (0.919–0.943)
Spence Children’s Anxiety Scale	0.940 (0.929–0.950)

Note: ^a^Item reflecting repetitive behavior was excluded from subscale

#### The parent-completed glasgow sensory questionnaire (GSQ-P)

The parent-completed GSQ (GSQ-P) contains 42 questions divided over 7 modality-specific subscales (visual, auditory, tactile, olfactory, gustatory, proprioceptive, and vestibular) and has been validated in 6–11 year-olds [16]. Questions are answered on a 5-point Likert scale (1 = Never, 2 = Rarely, 3 = Sometimes, 4 = Often, 5 = Always). We removed two items that clearly overlapped with the RRB domain of autistic function. One of these belonged to the auditory subscale (item 9: “Does your child like to listen to the same piece of music or part of a song over and over again?”) and one to the gustatory subscale (item 40: “Does your child like to eat the same foods most of the time?”). The internal consistencies of the modality subscales are shown in Table 1.

#### The developmental coordination disorder questionnaire (DCDQ’07)

The DCDQ’07 is a parent-completed questionnaire with 15 questions about motor skills, scored on a 5-point Likert scale (1 = Not at all like your child, 2 = A bit like your child, 3 = Moderately like your child, 4 = Quite a bit like your child, 5 = Extremely like your child). The cut-off for Indication of DCD was a score of 46 or less at age 5 to 7 years, 55 or less for 8 to 11 years, and 57 for 10 to 15 years [28]. We used the *reversed* total score as a measure of developmental motor dysfunction. See Table 1 for internal consistencies.

#### Spence’s children’s anxiety scale parent version (SCAS)

The Spence’s Children’s Anxiety Scale (SCAS) consists of 38 statements rated on a 4-point scale (0 = Never, 1 = Sometimes, 2 = Often, 3 = Always), with higher scores corresponding to higher anxiety [29]. It has been validated in children aged 6 to 17 years. The total score was skewed towards lower scores (median ± SD: 19 ± 16.3; range: 3 − 94; Shapiro-Wilk *p* = 2.6 × 10^–15^). Therefore, we used the log-transformed and z-scored SCAS score (ANX) as an outcome variable (Shapiro-Wilk *p* = 0.155). See Table 1 for internal consistency.

### Statistical analyses

Shapiro-Wilks tests were used to test for normality. Assumptions for linear regressions were checked by inspecting residuals, Q-Q plots, and variance inflation factors. Chi-squared tests and t-tests were used to test for sex differences in demographic and clinical variables (Table 2). Stochastic search variable selection (SSVS) was used to test for multivariable relationships between sensory predictors and QATs (ssvsforpsych.shinyapps.io/ssvsforpsych; 30). SSVS was chosen for its ability to identify the most important predictors from a large set of correlated variables, while accounting for model uncertainty [30]. All SSVS models included the seven sensory modality scores (visual, auditory, tactile, olfactory, gustatory, vestibular, proprioceptive), the motor score, sex, and age. Separate SSVSs were run for the dependent variables social QAT, communicative QAT, rigid QAT, and diagnosed ASC. The prior inclusion probability was set to 0.5, and the total number of iterations was 20,000 including 2,000 burn-in iterations. The SSVS was run twice for each dependent variable to confirm stability. All analyses were replicated in the subset of children that did not have a diagnosis of ASC. After identifying the sensory modalities most relevant (most robustly predictive) to QATs, a second set of SSVSs were run with selected sensorimotor predictors using ANX as the dependent variable. Linear regressions were used to test the effects of QATs or autism on ANX, including age, sex, and ADHD diagnosis in the null model. ADHD was included due to its high prevalence in the sample and its known association with sensory differences as well as anxiety disorders. Spearman’s correlation coefficients were calculated to test for bivariate relationships between the raw SCAS subscales and QATs.

## Results

Demographic details are shown in Table 2 and distributions of scores for the parent-report measures in Table 3. The sample of children was gender-balanced and largely from the United Kingdom. Clinical autism was present in 12.8% of the children, autism in a first-degree relative was reported for 8.9%, and 21% of children exceeded the AQ screening cut-off of 76 [27]. No one reported intellectual disability. Most informants (95%) were biological mothers, 0.4% were adoptive mothers, and 5% were other female caregivers (e.g., grandmother with custody, stepmother, or legal guardian).

**Table 2:** Demographic details

	Total	Male	Female	Test statistic	*p*-value
N	257	130	127		
Age (mean ± SD)	8.43 ± 1.82	8.59 ± 1.75	8.27 ± 1.88	t = − 1.399	*p* = 0.163
Country of residence					
United States	10.1%	10.0%	10.2%	Χ^2^ = 0.004	*p* = 0.950
United Kingdom	89.9%	90.0%	89.8%		
Neurodevelopmental conditions					
Autism	12.8%	14.6%	11.0%	Χ^2^ = 0.741	*p* = 0.389
Attention deficit/hyperactivity disorder	10.1%	12.3%	7.9%	Χ^2^ = 1.389	*p* = 0.239
Tourette’s syndrome/tic disorder	1.6%	3.1%	0.0%	Χ^2^ = 3.969	*p* = 0.046*
Specific learning disorder	1.6%	1.5%	1.6%	Χ^2^ = 5.5 × 10^–4^	*p* = 0.981
Communication disorder	1.2%	1.5%	0.8%	Χ^2^ = 0.314	*p* = 0.575
Intellectual disability	0.0%	0.0%	0.0%	—	—
Developmental coordination disorder	0.0%	0.0%	0.0%	—	—
Stereotypic movement disorder	0.0%	0.0%	0.0%	—	—
Autism in informant (if biological mother)					
No	78.6%	80.0%	77.2%		
Yes	3.9%	3.8%	3.9%	Χ^2^ = 0.432	*p* = 0.806
Suspected	12.1%	13.4%	10.8%		
Autism in other first-degree relative					
No	82.1%	86.2%	78.0%		
Yes	5.4%	5.4%	5.5%	Χ^2^ = 4.606	*p* = 0.100
Suspected	7.0%	3.8%	10.2%		
AQ score above cut-off (≥ 76)	21.0%	22.3%	19.7%	Χ^2^ = 0.266	*p* = 0.606

Note: *, *P* < 0.05

**Table 3:** Descriptive questionnaire data

	Mean ± SD	Range	Possible range
**AQ-Child**			
Total score	59.0 ± 25.3	15–128	0–141
Social QAT	10.2 ± 6.3	1–30	0–30
Communicative QAT	12.0 ± 6.5	0–29	0–30
Rigid QAT	13.9 ± 6.1	2–30	0–30
**Glasgow Sensory Questionnaire**			
Total score	81.5 ± 27.7	44–171	42–210
Visual	10.4 ± 4.3	6–27	6–30
Auditory	14.7 ± 5.3	6–29	6–30
Tactile	11.8 ± 4.5	6–28	6–30
Olfactory	11.4 ± 4.1	6–26	6–30
Gustatory	12.3 ± 4.4	6–27	6–30
Vestibular	9.8 ± 4.1	6–25	6–30
Proprioceptive	11.7 ± 5.4	6–28	6–30
**Developmental Coordination Disorder Questionnaire (DCDQ)**			
Total score	56.3 ± 13.8	19–75	15–75
Control during Movement	22.9 ± 5.6	6–30	6–30
Fine motor/handwriting	16.1 ± 4.6	4–20	4–20
General coordination	17.3 ± 5.3	5–25	5–25
**Spence Children’s Anxiety Scale (SCAS)**			
Total score	22.9 ± 16.3	3–94	0–114
Panic attack/agoraphobia	2.1 ± 3.6	0–23	0–27
Separation anxiety	5.3 ± 3.8	0–18	0–18
Physical injury fears	4.0 ± 2.6	0–12	0–15
Social phobia	5.2 ± 3.9	0–17	0–18
Obsessive compulsive disorder	2.1 ± 2.9	0–15	0–18
Generalized anxiety disorder	4.4 ± 3.4	0–17	0–18

*Note*. The AQ consisted of 47 items (scored 0–3; high score = more QATs), the Glasgow Sensory questionnaire of 42 items (scored 1–5; high score = more difficulties), the DCDQ of 15 items (scored 1–5; high score = better coordination) and the SCAS of 38 items (scored 0–4; high score = more anxiety). Please note that these raw scores are shown to illustrate the sample, whereas the main regression analyses instead used the reversed DCDQ score and log-transformed z-scored SCAS (see Methods)

### Specific sensorimotor predictors of QATs and ASC

**Fig. 1:**
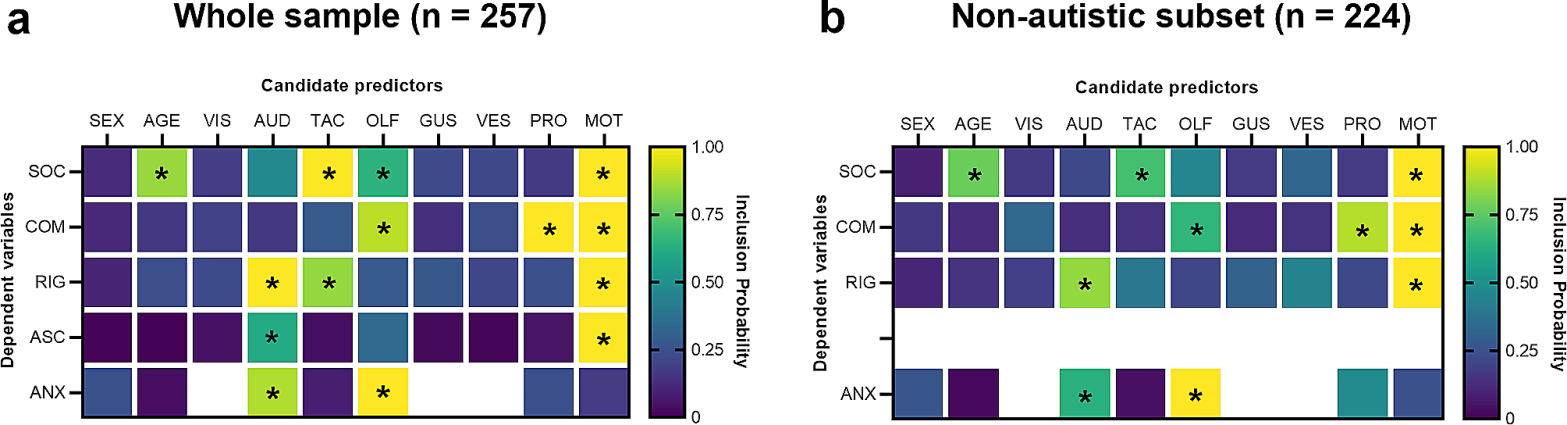
Marginal inclusion probabilities for sensorimotor predictors of autistic traits, autism, and anxiety. Predictors were age, sex, scores for the seven sensory modalities (Glasgow Sensory Questionnaire Parent version) and motor coordination scores (Developmental Coordination Disorder Questionnaire). Stochastic Search Variable Selection (SSVS) was run for each Quantitative Autistic Trait (QAT) domain (SOC, social QAT; COM, communicative QAT; RIG, rigid QAT), and diagnosed autism (ASC, autism spectrum conditions). Selected predictors were then used for an SSVS with the anxiety score (ANX) as the dependent variable (Spence’s Children’s Anxiety Scale Parent version, log-transformed total score). The same analyses were run in the whole sample (**a**) and the non-autistic subset (**b**). Asterisks denote inclusion probabilities above 0.5. Abbreviations: AUD, auditory; GUS, gustatory; MOT, motor; OLF, olfactory; PRO, proprioception; TAC, tactile; VES, vestibular; VIS, visual

We first aimed to replicate the results from the previous study in adults, which reported modality-specific associations between sensory symptoms and QATs [26]. We used SSVS to select the most robust sensorimotor predictors of individual QATs, interpreting high marginal inclusion probabilities (MIPs) as indicative of a high likelihood of relevance to QATs and/or ASC. In addition to the seven sensory scores, we included the DCDQ motor score as a predictor to correct for motor components of the GSQ-P proprioception score. This analysis identified motor dysfunction as a robust predictor of social, communicative, and rigid QATs (Fig. 1a, rows 1–3) and this replicated when autistic children were excluded (Fig. 1b). Identical to the previous study in adults [26], we found high MIPs for tactile symptoms as a predictor of social QAT, proprioceptive symptoms for communicative QAT, and auditory symptoms for rigid QAT (Fig. 1a-b). In addition, olfactory symptoms were selected as a predictor of communicative QATs (Fig. 1a-b). Less robust relationships existed for social QATs versus olfactory symptoms and rigid QATs versus tactile symptoms (Fig. 1a), but these did not remain above threshold when autistic children were removed (Fig. 1b). Auditory symptoms and motor difficulties were most predictive of diagnosed autism in this sample (Fig. 1a, row 4). Based on the above findings, we excluded visual, gustatory, and vestibular scores from further analyses.

### Heightened anxiety in high-QAT children

ANX was strongly predicted by diagnosed ASC (Adjusted R^2^ = 0.171, F_3,253_ = 18.545, *p* = 6.6 × 10^–11^; t = 7.220, *p* = 6.1 × 10^–12^) as well as by social QAT (standardized β = 0.555, t = 10.634, *p* = 4.6 × 10^–22^), communicative QAT (standardized β = 0.568, t = 10.502, *p* = 1.2 × 10^–21^), and rigid QATs (standardized β = 0.629, t = 12.789, *p* = 3.4 × 10^–29^) (see Table 4 for model fits; models corrected for age, sex, and ADHD). All SCAS subscales were significantly correlated with the individual QATs (Spearman’s correlations; Table 5).

**Table 4:** Regression models with social, communicative, and rigid QATs as predictors of anxiety

	Adjusted *R*^2^	F	df	*p*
Social QAT	0.319	31.000	4, 252	5.4 × 10^–21^
Communicative QAT	0.314	30.282	4, 252	1.4 × 10^–20^
Rigid QAT	0.402	43.998	4, 252	5.5 × 10^–28^

Note: All models were corrected for age, sex, and ADHD. Social QAT were measured with the AQ-Social Skills subscale; Communicative QAT were measured with the AQ-Communication subscale; and Rigid QAT were measured with the AQ-Attention switching subscale. Variables were z-scored

**Table 5:** Spearman’s correlation coefficients (r) between QATs and SCAS subscales

	SOC	COM	RIG
Panic attack/agoraphobia	0.444*	0.430*	0.548*
Separation anxiety	0.408*	0.403*	0.514*
Physical injury fears	0.349*	0.296*	0.328*
Social phobia	0.447*	0.366*	0.416*
Obsessive compulsive disorder	0.356*	0.420*	0.510*
Generalized anxiety disorder	0.363*	0.437*	0.519*

Note: *, *p* < 1 × 10^–6^. QATs were z-scored. SCAS subscale scores were not transformed or normalized

### Specific sensorimotor predictors of anxiety

Sensorimotor symptoms (auditory, olfactory, tactile, proprioceptive, and motor), age and sex were entered as predictors in a second set of SSVS analyses with ANX as the dependent variable (Fig. 1, bottom rows). Olfactory and auditory dysfunction showed the highest MIPs for ANX (1.00 and 0.88, respectively), whereas age, sex, tactile, proprioceptive, and motor dysfunction showed low MIPs (Fig. 1a). Similar results were found when autistic children were excluded from the analysis (Fig. 1b; MIP = 1.00 for olfactory, 0.63 for auditory, and < 0.5 for other variables).

Finally, linear regressions were used to estimate the effect sizes of the associations between auditory or olfactory symptoms with anxiety, correcting for age, sex, and total QAT. For auditory symptoms, the standardized β coefficient was 0.322 [95% CI: 0.189, 0.455] (Adjusted R^2^ = 0.429, F_4,252_ = 49.067, *p* = 1.7 × 10^–30^). For olfactory symptoms, the standardized β was 0.354 [95% CI: 0.239, 0.469] (Adjusted R^2^ = 0.547, F_4,252_ = 54.761, *p* = 3.5 × 10^–33^). When the effect sizes were estimated for auditory and olfactory symptoms independently, by including both together, significant independent effects were observed for the two sensory modalities (standardized β = 0.192 for auditory symptoms and 0.285 for olfactory symptoms; adjusted R^2^ = 0.470, F_5,251_ = 46.368, *p* = 7.9 × 10^–34^).

## Discussion

We used a pediatric sample to replicate modality-specific associations between sensory symptoms and QAT domains previously reported in two adult samples [26], and expanded findings to the motor domain. The specific phenotypic associations mirroring the adult study were (1) tactile symptoms and social skills, (2) proprioceptive symptoms and communicative differences, and (3) auditory symptoms and rigid personality. The proprioceptive/communicative association remained in this study despite including a motor function scale, indicating a role for both proprioception and motor skills. Auditory and olfactory symptoms were robustly and independently predictive of heightened anxiety, also when correcting for other autistic characteristics, suggesting that these modalities may be particularly relevant for mental health. The striking similarities as well as the differences between our findings in children and previous findings in adults [26] will be discussed below.

### Auditory processing, rigid personality and anxiety

One main difference in children compared to adults was that auditory function was a more specific predictor for the rigid QAT domain whereas in adults it was broadly predictive of the full autistic phenotype. Further, olfactory symptoms appeared much more dominant in children than in adults. Our interpretation of these differences is limited by uncontrolled factors associated with the use of two different QAT instruments and parent- versus self-report. The constructs measured by the Social Skills and Communication subscales of AQ-Child may not be entirely equivalent to those measured by the BAPQ’s Aloofness and Pragmatic language subscales, and scores may be affected by life experience. This can only be resolved by longitudinal work.

The specific association between rigid personality and auditory symptoms seen in the current study might reflect a real phenotypic cluster, as it has held up across two adult samples (*n* = 252 + 268) and this pediatric sample (*n* = 277). Autism studies have generally used instruments that pool sensory modalities and/or QAT subdomains, but Schulz and Stevenson [31] reported that high scores on the Auditory Hypersensitivity subscale of the Adult/Adolescent Sensory Profile were correlated with all QAT domains in an adult sample. In addition, general population studies have reported associations between noise sensitivity and low extraversion (similar to social QAT) and low openness to experience (similar to rigid QATs) [32, 33]. Aside from associations between these stable traits, there are probably environmental contributions. For example, behavioral rigidity may be a coping mechanism to minimize auditory stimulation, and noise sensitivity might worsen in the presence of stress. A rigid intolerance of uncertainty is in and of itself strongly associated with anxiety and stress [11, 34], and autonomic reactivity is associated with hyperreactivity to auditory stimuli [35]. Effects of acute auditory stressors on cognitive flexibility have also been reported [36]. The contribution of rigid and auditory symptoms to anxiety in the current study suggests that there are dimensional relationships that may be addressed at an early age in children with high rigidity. Further studies may also elucidate which type of auditory symptoms are most relevant to anxiety, because the GSQ subscale addresses several auditory functions, including aversion, seeking and speech perception [24].

### The link between olfactory differences and anxiety

The olfactory system connects heavily and directly to limbic structures, and altered olfactory reactivity is associated with autism as well as anxiety [24, 37, 38]. Children with autism showed altered odor awareness [39] and GSQ olfactory scores were elevated in individuals with high total QAT [16, 24]. The weaker relationship with QATs in our previous adult study compared to the current pediatric sample may reflect age differences, but it may also be confounded by differences in the internal reliability of the olfactory subscale (α = 0.730 in this study; 0.49–0.52 in Bang and Igelström, 2023), as low reliability of a construct attenuates correlations with it. Multiple autism studies have examined objective olfactory ability, such as discrimination and identification, but the results have been heterogeneous and possibly moderated by age [40]. The affective response to odors as measured by self-report appears more robustly altered in autism than do earlier stages of olfactory processing. Both olfactory and auditory symptoms were perceived by autistic adults to cause anxiety, rather than anxiety being the cause of sensory symptoms [41], but experimental studies on causal relationships are still lacking.

### Proprioceptive and motor function

The previously observed link between proprioceptive function and communicative QATs [26] were replicated in this study. Importantly, proprioceptive symptoms remained predictive of communicative QATs despite controlling for motor dysfunction, supporting a role for the proprioceptive sense. However, the nature of this relationship is difficult to interpret without more specific studies. Proprioceptive symptoms, such as decreased awareness of the body’s position in space, are common in individuals with developmental coordination disorder, and early motor deficits have previously been associated with atypical development of communication or language [42, 43]. Developmental motor symptoms were broadly associated with all three QATs in the current study, consistent with the common presentation of coordination difficulties in autism. Since motor activity is essential for both verbal and nonverbal communication, it is possible that early deficits prevent typical development of these higher-level functions.

### Tactile differences and social aloofness

Replicating previous associations between tactile sensitivity and social QAT [26], our results reinforce the notion of altered social touch in autism [44–46] and add relative specificity for the social interaction domain as well as a dimensional relationship that extends to subclinical levels. Consistent with this, a relationship between aversion to social touch and total QATs has previously been reported [47]. The role of touch is thought to be crucial for early social development, and tactile sensitivity might be one of the driving forces for altered social development in autistic individuals [45]. Our results add support for a strong linear relationship between autistic-like tactile symptoms and social skills in both children and adults with and without ASC.

### Limitations

The most significant limitation of this study was the use of parent-rated questionnaire subscales to quantify specific symptoms, rather than clinical evaluation or objective measures. In addition, the impact of potential measurement errors associated with parent-report cannot fully be estimated, even though the parent-rated SCAS were previously shown to be significantly correlated with the child-rated version, with the highest agreement for separation anxiety and lower for more internalizing anxiety components [29, 48].

While the GSQ, DCDQ and AQ have all been validated in terms of their reliability and construct validity, there are notable weaknesses. In particular, the GSQ modality subscales have shown relatively low reliability in some studies [16, 24, 26]. However, the current study found good subscale reliabilities (Table 1), strengthening our interpretations. The GSQ modality subscales quantify a mixture of symptoms, comprising sensory sensitivity, sensory seeking, and sensory under-responsivity. It will be necessary to study each domain in greater detail to fully understand the roles and mechanisms or different types of sensory symptoms. The current study suggests relevant modalities for such studies. Another limitation is that we recruited female caregivers exclusively via the Prolific platform, causing a sampling bias. The size and nature of this bias is largely unknown since we did not ask for detailed socio-economic information. While we could reach more diverse populations than our local environment provides, there is relatively low ethnic diversity on Prolific, favoring white people residing in the US or UK [49, 50]. Our sample was dominated by parents from the UK, limiting generalizability.

13% of the children were diagnosed as autistic, and 21% had AQ scores above a suggested screening threshold of 76 points (Table 2). This relatively low proportion of autistic individuals (compared with study designs that aim to balance the groups), together with the lack of clinical evaluation, limits the utility of group comparisons. Our focus of the study was instead the dimensional nature of the broader autistic phenotype, and we used an approach that is most relevant to polygenic forms of autism. We acknowledge that QATs are not synonymous to ASCs [51] and suggest that the results are applicable to the populations as a whole, with likely relevance to clinical ASCs. We also did not quantify levels of functioning or need for support, beyond diagnosed conditions. This limits our knowledge of the sample and therefore the possibility to make clinical interpretations.

## Conclusions

This study, together with our previous report [26], have established the following specific dimensional associations between classical QATs and sensory symptoms: (1) Social and Tactile, (2) Communicative and Proprioceptive, and (3) Rigid and Auditory. The sensory symptoms most predictive of high anxiety were in the auditory and olfactory domains. The study further suggests that symptoms of developmental coordination disorder are highly linearly correlated with classical QATs, but less likely to be associated with anxiety. We suggest that differences in auditory and olfactory processing may be especially relevant for intervention studies, as potential contributors to – and/or consequences of – poor mental health in children with high levels of QATs.

## Data Availability

The dataset supporting the conclusions of this article is available in the Open Science Framework repository, [unique persistent identifier and hyperlink to dataset(s) in http://format].

## Abbreviations

ADHD: Attention deficit/hyperactivity disorder
ANX: z-scored log-transformed score on the Spence’s Children’s Anxiety Scale
ASC: Autism spectrum conditions
AQ: Autism Spectrum Quotient
BAPQ: Broad Autism Phenotype Questionnaire
CATI: Comprehensive Autistic Traits Questionnaire
DCD: Developmental coordination disorder
DCDQ: Developmental Coordination Disorder Questionnaire
GSQ: Glasgow Sensory Questionnaire
RRB: Rigid/repetitive behaviors
SCAS: Spence’s Children’s Anxiety Scale
SSVS: Stochastic search variable selection
QAT: Quantitative autistic traits
